# A two-colour multiplexed lateral flow immunoassay system to differentially detect human malaria species on a single test line

**DOI:** 10.1186/s12936-019-2957-x

**Published:** 2019-09-18

**Authors:** Jinsu Kim, Xiangkun Elvis Cao, Julia L. Finkelstein, Washington B. Cárdenas, David Erickson, Saurabh Mehta

**Affiliations:** 1 Division of Nutritional Sciences, Cornell University, Ithaca, NY USA; 2 Sibley School of Mechanical and Aerospace Engineering, Cornell University, Ithaca, NY USA; 3 Institute for Nutritional Sciences, Global Health, and Technology, Cornell University, Ithaca, NY USA; 4 Escuela Superior Politécnica del Litoral, Guayaquil, Ecuador

**Keywords:** Malaria, Diagnostics, Screening, Point of care, Multiplex

## Abstract

**Background:**

Malaria continues to impose a tremendous burden in terms of global morbidity and mortality, yet even today, a large number of diagnoses are presumptive resulting in lack of or inappropriate treatment.

**Methods:**

In this work, a two-colour lateral flow immunoassay (LFA) system was developed to identify infections by *Plasmodium *spp. and differentiate *Plasmodium falciparum* infection from the other three human malaria species (*Plasmodium vivax*, *Plasmodium ovale*, *Plasmodium malariae*). To achieve this goal, red and blue colours were encoded to two markers on a single test line of strips, for simultaneous detection of PfHRP2 (red), a marker specific for *P. falciparum* infection, and pLDH (blue), a pan-specific marker for infections by all species of *Plasmodium*. The assay performance was first optimized and evaluated with recombinant malarial proteins spiked in washing buffer at various concentrations from 0 to 1000 ng mL^−1^. The colour profiles developed on the single test line were discriminated and quantified: colour types corresponded to malaria protein species; colour intensities represented protein concentration levels.

**Results:**

The limit of detection (the lowest concentrations of malaria antigens that can be distinguished from blank samples) and the limit of colour discrimination (the limit to differentiate pLDH from PfHRP2) were defined for the two-colour assay from the spiked buffer test, and the two limits were 31.2 ng mL^−1^ and 7.8 ng mL^−1^, respectively. To further validate the efficacy of the assay, 25 human whole blood frozen samples were tested and successfully validated against ELISA and microscopy results: 15 samples showed malaria negative; 5 samples showed *P. falciparum* positive; 5 samples showed *P. falciparum* negative, but contained other malaria species.

**Conclusions:**

The assay provides a simple method to quickly identify and differentiate infection by different malarial parasites at the point-of-need and overcome the physical limitations of traditional LFAs, improving the multiplexing potential for simultaneous detection of various biomarkers.

## Background

Over 40% of world’s population live in malaria-endemic areas, and malaria is found in more than 100 countries in Africa, Latin America, the Caribbean, Southeast Asia, the Eastern Mediterranean, the Western Pacific, and parts of Europe [1]. According to the recent World Malaria Report by the World Health Organization (WHO), an estimated 219 million new malaria cases, and 435,000 new deaths occurred worldwide in 2017 [2]. Despite increasing malaria control measures, malaria infection remains a global threat for millions of children, especially for those in sub-Saharan Africa [3].

Early diagnosis and prompt, appropriate treatment is essential for improving patient outcomes, preventing overuse of malarial drugs, and minimizing development and spread of anti-malarial drug resistance [4]. The current practices for malaria diagnosis include clinical assessment, microscopic evaluation of peripheral blood smears, and the use of rapid diagnostic tests (RDTs) [5]. RDTs have been used widely in malaria-epidemic countries due to their simplicity, low cost and the ability to provide an early diagnosis [6]. Most RDTs for malaria diagnosis are based on a lateral flow immunoassay (LFA), with pre-coated antibodies on test lines. These antibodies will bind to malaria antigens, such as parasite lactate dehydrogenase (pLDH), *Plasmodium falciparum* histidine-rich protein2 (PfHRP2), and parasite aldolase (pAldo) [7, 8]. The commercially available RDTs are often manufactured in the form of three lines (e.g., two test lines, and a control line) on the LFA strip for the multiplexed detection. For instance, PfHRP2/(Pan) pLDH rapid test enables simultaneous detection for PfHRP2 and pLDH [9]. PfHRP2 detection is specific to *P. falciparum* only, and pLDH pan-specific to all *Plasmodium* species. The concurrent detection of PfHRP2 and pLDH allows the discrimination of *P. falciparum* infection from the other three human malaria species (i.e., *P. vivax*, *P. ovale*, *P. malariae*).

Severe malaria leads to significant mortality and is mainly attributed to *P. falciparum* infection, particularly when treatment is delayed [10]. Patients suffering from severe malaria should be hospitalized and treated intensively with intravenous anti-parasite drugs.

In addition to malaria species and severity, the treatment approach of malaria also depends on special risk groups, such as infants and pregnant women [11]. For children with glucose-6-phosphate dehydrogenase deficiency (G6PD), WHO recommended testing G6PD before prescription of anti-malaria drug to ensure safe administration of primaquine for preventing relapse of *P. vivax* and *P. ovale* malaria [12]. For malaria with coincidental pregnancy, commercial combo RDTs allows detection of human chorionic gonadotropin (hCG) with an additional test line on the LFA strip. Measuring other biomarkers such as ferritin, and angiopoietin-1 and -2 levels help further diagnose malaria complication of severe anaemia and cerebral malaria, respectively [13–15]. Thus, it is anticipated that the next generation of RDTs should have multiplexing potential for detecting multiple biomarkers simultaneously, to provide patients with more health data. However, multiplexing in traditional LFAs usually brings more test lines, which is confined to the spatial and physical limitations of the strip. This is further complicated by the uncertainty of flow changes when passing through multiple lines [16].

**Fig. 1:**
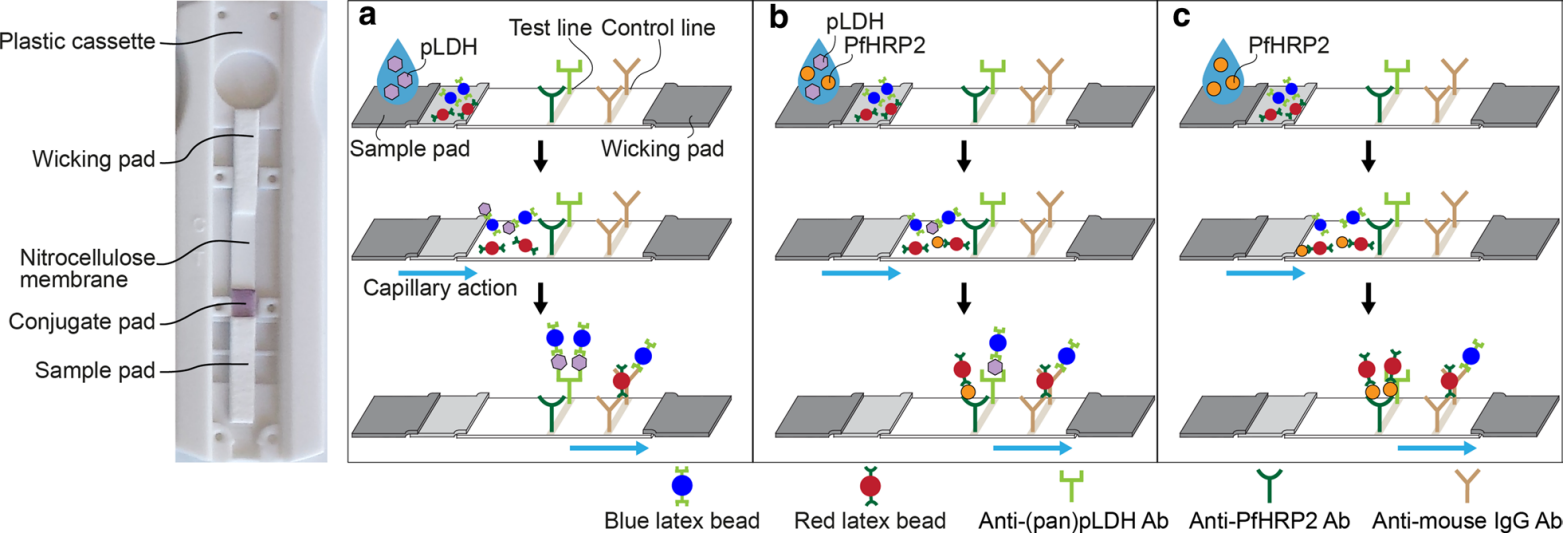
Schematic of two-colour lateral flow assay. Mixture antibodies to (pan)pLDH and PfHRP2, and antibodies to mouse IgG were modified on nitrocellulose to generate test and control lines, respectively. The malaria antigens of individual pLDH, HRP2 or both were dispensed onto a sample pad of strips that were then washed in buffer. The blue and red latex particles enabled to capture pLDH and PfHRP2 at a conjugate pad, respectively. Then, antigens bound to latex particles were transported through the strip and sandwiched at the test lines. The different colours were developed on the test regions corresponding a type of antigens and concentrations. Schematic details of the test regions showed the test lines with (**a**) the blue colour for pLDH detection only, (**b**) mixture colour of blue and red for simultaneous detection, and (**c**) red colour for PfHRP2 detection only

Here, a quantitative, multiplexing lateral flow immunoassay using two-colour latex particles to overcome current limitations of RDTs is reported. The red and blue latex particles were first functionalized with antibodies to PfHRP2 and (pan) pLDH, respectively. In the LFA, nitrocellulose membranes were pre-coated with one test line and one control line. The test line contains a mixture of antibodies to PfHRP2 and (pan) pLDH, and the control line contains antibodies to mouse IgG, as shown in Fig. 1. The assay was first tested with recombinant malaria antigens of known concentrations spiked into washing buffer. Then the assay was validated against gold standard approaches (ELISA and microscopy) with human clinical samples. The two-colour LFA showed a promising approach of using a single test line for multiplexed differential detection. It is anticipated that the assay could be further extended for multiplexing, with multi-colour conjugation and improved colour discrimination algorithm.

## Methods

### Functionalization of latex particles

The 400 nm blue and red latex particles (Innova Biosciences, #1000-0100, #1002-0100) were functionalized with anti-(pan)pLDH antibodies (MyBioSource, #MBS313259), and anti-PfHRP2 antibodies (MyBioSource, #MBS313020), respectively, according to the manufacturer’s instruction.

### Preparation of LFA strips

To prepare the test and control lines, a mixture of 1 mg mL^−1^ anti-(pan)pLDH antibody (MyBioSource, #MBS313265) and 1 mg mL^−1^ anti-PfHRP2 antibody (MyBioSource, # MBS312993) in PBS, and 10 mg mL^−1^ anti-mouse IgG antibody (antibodies-online, #ABIN458529) in PBS were dispensed to the nitrocellulose membrane (Millipore, HF180MC1000), respectively, with a dispenser (ClaremontBio, 07.711.01) and a syringe pump (KD Scientific, 78-8200), followed by being dried overnight and stored at 37 °C until use. One cm × 12 cm glass fibre conjugate pad (Millipore, GFDX103000) was fully immersed in 800 μL conjugate buffer, with a mixture of 0.05% (v/v) blue and 0.05% (v/v) red latex particles in 2 mM borate buffer with 5% sucrose, followed by being dried overnight and stored at 37 °C. The conjugate pad, sample pad (Millipore, CFSP203000), and absorbent pad (Millipore, CFSP203000) were assembled onto the adhesive parts of the nitrocellulose membrane. Then the LFA was cut to 4 mm × 6 cm in dimension and was caged in the plastic cassette.

### Dilution of malarial proteins

The LFA performance was demonstrated by detecting recombinant malaria antigens, including pLDH (MyBioSource, #MBS319848), and PfHRP2 (MyBioSource, #MBS319418). A series of twofold dilutions of each protein of pLDH, and PfHRP2 in washing buffer (1× TBS with 1% BSA, 1% Tween 20) were prepared at various concentrations from 3.9 to 1000 ng mL^−1^. For simultaneous detection, pLDH and PfHRP2 antigens were mixed at the ratio of 1 to 6, followed by twofold serial dilution with washing buffer.

### LFA test and image analysis

Thirty microliters of the sample was dispensed on a sample pad of the LFA strip, followed by being washed by 70 μL washing buffer to allow the sample liquid to flow through the full length of the nitrocellulose membrane. After 15 min, images of the strips were acquired and saved using iPad Air2 which was equipped with a 3D printed accessory iPad case. To bypass the challenges of naked-eye detection, a plug-in command called ‘Color Profiler’ in ImageJ software (https://imagej.nih.gov/ij/plugins/color-profiler.html) was employed for obtaining colour profiles in the test strip images. The region of interest (ROI) was selected on the images where both test and control lines were included using rectangular selections on the ImageJ menu bar. The same ROI was applied to all acquired images. The red and blue colour intensity profiles along with the distance of the strip were plotted, and the peak areas at the test regions were calculated using Simpson’s 3/8 rule.

### Clinical samples

To further validate the performance of the assay, 25 clinical research samples in whole blood form were tested: 15 malaria negative samples, 5 *P. falciparum* positive samples, and 5 *P. vivax* positive samples. All samples were purchased from Discovery Life Sciences and results were confirmed by microscopy. For the 5 *P. falciparum* malaria whole blood samples, the microscopy test results for DLS17-026025, DLS17-049460, DLS17-049463, DLS16-71770 and DLS17-049468 were 112,216/μL, 128,848/μL, 104,168/μL, 70,117/μL and 91,246/μL, respectively. For the 5 *P. vivax* malaria whole blood samples, the microscopy test results for DLS15-35471, DLS17-048442, DLS17-048486, DLS17-049457 and DLS17-048476 were 48,620/μL, 34,285/μL, 32,592/μL, 116,800/μL and 34,760/μL, respectively. All the 10 malaria positive samples were diluted by 20 times before testing. For the 15 malaria negative whole blood samples, they were confirmed by microcopy results and they included: DLS17-037032, DLS17-037035, DLS17-037040, DLS17-037043, DLS17-037221, DLS17-037223, DLS17-037224, DLS17-037225, DLS17-037226, DLS17-037227, DLS17-037228, DLS17-037230, DLS17-037231, DLS17-037234 and DLS17-037235.

The samples were also tested using commercially available ELISA kits (Cellabs, #Quantimal pLDH CELISA, and #Quantimal Pf-HRP2 CELISA) to get quantitative results to evaluate the performance of the LFA strips.

## Results

### Principle of two-colour LFA in the multiplexed detection of malarial antigens at a single test line of the LFA strip

In the LFA, when the sample liquid is dispensed on a sample pad and flows to the conjugate pad, the blue and red latex particles capture pLDH and PfHRP2 antigens, respectively. The antigens bound to the latex particles are subsequently transported through the strip, and are detected at the test line where a mixture of detection antibodies to (pan)pLDH and PfHRP2 are functionalized (Fig. 1). The change in the colour profiles developed on the test region corresponds to the number of the captured blue and red latex particles.

**Fig. 2:**
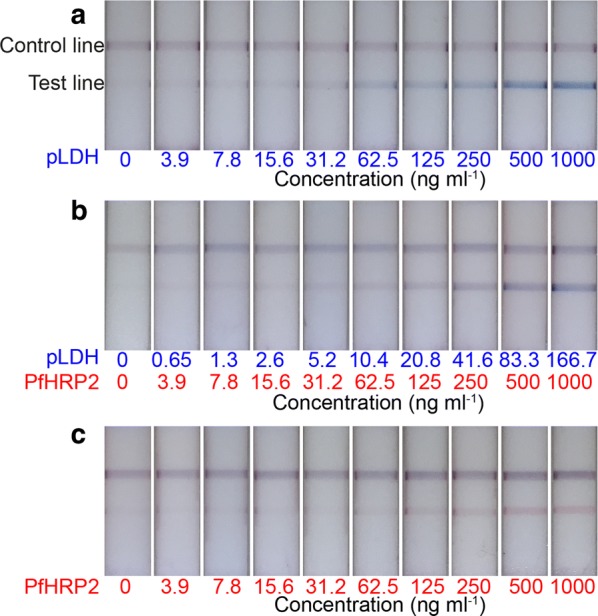
Representative test strips of the two-colour LFA. Distinct colours were observed at the test lines, corresponding a type of antigens, such as **a** blue test lines for pLDH detection only, **b** mixture colour of test lines for simultaneous detection, and **c** red test lines for PfHRP2 detection only. The mixture colour at control lines indicated the red and blue latex particles migrated along the length of the strip

The performance for the two-colour LFA was first demonstrated by detecting recombinant malarial antigens spiked in the washing buffer at various concentrations. Three different diagnostic scenarios were tested: pLDH only, PfHRP2 only, and concurrent pLDH-PfHRP2 infection. For the co-detection, pLDH and PfHRP2 was mixed at a molar ratio of 1 to 6, to mimic clinical samples of *P. falciparum* infection [17]. Fifteen minutes after sample dispensation followed by buffer wash, the colour profiles on the test regions were recorded. When the sample contained pLDH only, blue test lines were observed (Fig. 2a). In the simultaneous detection, the test region showed a mixture colour of blue and red (Fig. 2b). For PfHRP2 only samples, test lines turned red (Fig. 2c). The control lines on all LFA strips showed a mixture colour of blue and red, indicating both red and blue latex particles were transported through the strips.

### Features in colour profiles of LFA strips

**Fig. 3:**
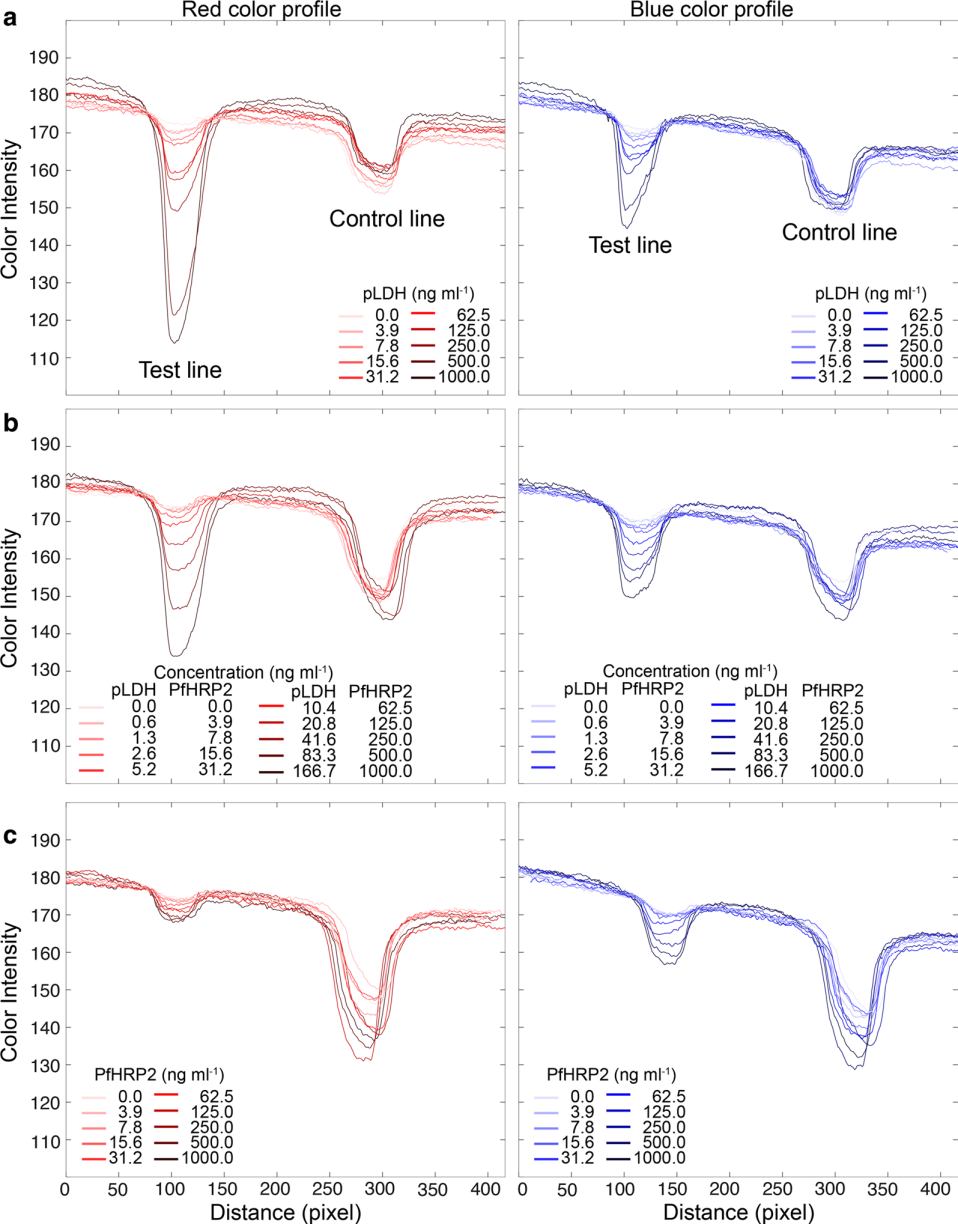
Red and blue intensity profiles of test strips. RGB colour profiles were obtained from the strip images from Fig. 2. For simplicity, red and blue intensities were represented, except for the green intensity. **a** When blue colours appeared on the test lines, red intensity was more decayed than blue intensity. This trend was because the background of the white strip retained high RGB values. **b** When mixture colour appeared at the test lines, red and blue peaks were generated corresponding to the concentration of antigens. **c** For PfHRP2 detection only, the blue peaks were more decayed than red peaks, in reverse observation from **a**

To implement the quantitative and qualitative method in the assay, the intensity profiles of the LFA strips were analysed. The images of the strips were acquired using an 8-megapixel rear-facing camera of an iPad Air 2 under the same white LED lighting conditions. The distance between the test line and control line was about 200 pixels, and width of the line was about 50 pixels in the images. To obtain the RGB color profiles, the images were opened using ImageJ software and executed “Color Profiler” command. For simplicity, only the red and blue intensity profiles were analysed, since green intensity profiles did not significantly affect the red and blue colour discrimination, and provided an auxiliary value in colour images. The nitrocellulose membrane of the test strip was white, resulting in high background intensities. The colours with contrast at the test and control lines generated the peaks decayed from the background intensities (Fig. 3).

Two intriguing features in the colour profiles were observed. First, when the blue test lines appeared on the strips, the red intensity peaks were more decayed than blue peaks in the colour profiles. This is because the blue colour retained relatively higher blue pixel values than red values. Figure 3 shows the red and blue intensity profiles of the strips extracted from images in Fig. 2. For pLDH detection only where strong blue test lines were observed, the red intensities were significantly decayed from top background intensities, more than blue peaks (Fig. 3a). In the same context, PfHRP2 detection with apparent red test lines in the images generated the lower blue peaks than red peaks (Fig. 3c).

The other feature in the colour profiles was the non-specific colour peaks in Fig. 3a (blue intensity) and Fig. 3c (red intensity) that were not associated with colour development by latex particles. Non-specific binding or cross-reactivity on the test lines was not observed by naked eyes. Indeed, Fig. 2 showed clear distinction of colours for each detection mode. However, the non-specific intensity peaks were developed by the image contrast. As latex particles were accumulated at the test lines, the darkness increased, resulting in decreasing RGB values. Thus, all intensity peaks in Fig. 3 were not from the pure colours but were affected by image contrast. It was not easy to decouple the contrast and pure colour from images. However, a simple correlation function was established by calculating the ratio of the red to blue decay areas to discriminate the colour type. This was addressed and is discussed in the next section.

### Limit of detection and limit of colour distinction

To further analyse the strips, avoid subjectivity, and confirm visual limit of detection, decay areas of red and blue peaks were calculated from Fig. 3. To calculate the peak areas, peak alignment was first performed on the background intensity. And then Simpson’s 3/8 rule was applied to the aligned peaks for the numerical integration to calculate areas.

**Fig. 4:**
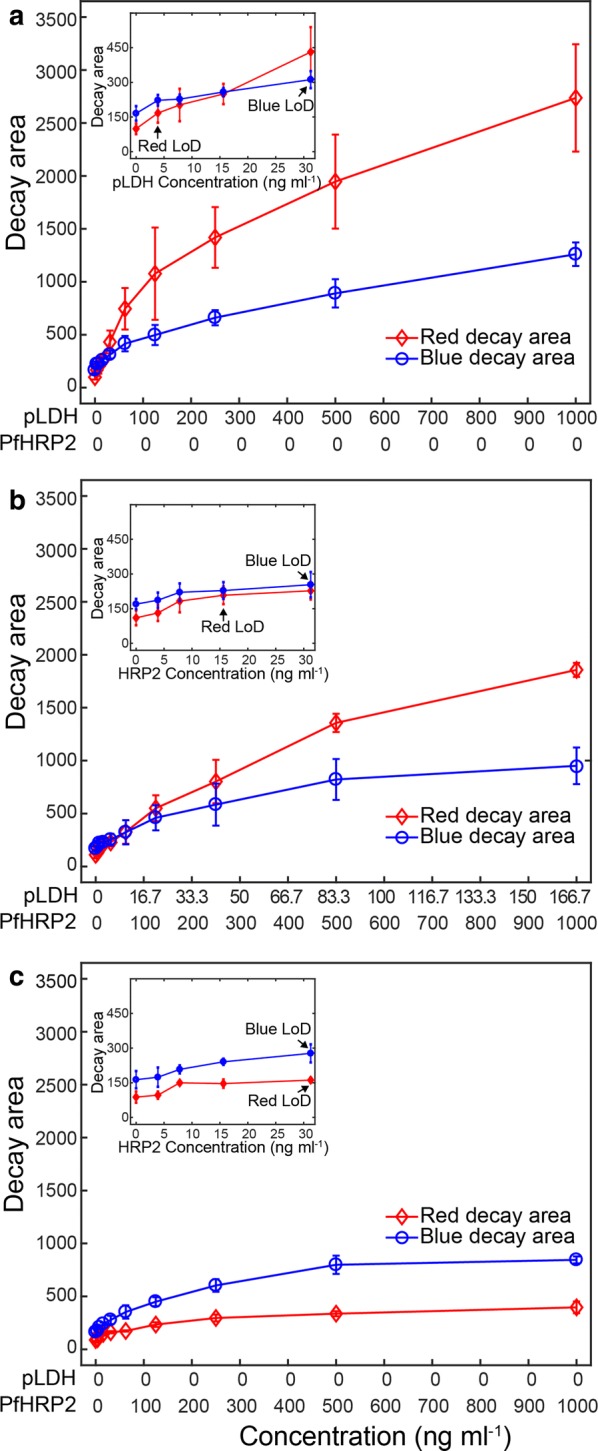
Calculation of red and blue decay areas at the test lines. The red and blue decay areas were calculated from Fig. 3 for **a** pLDH detection only, **b** simultaneous pLDH and PfHRP2 detection, and **c** PfHRP2 detection only. The different degrees of decay areas between the red and blue intensities were observed as a function of sample types and concentrations. The inserted graphs were zoomed in at lower concentrations. The LoD to distinguish from non-target samples was 31.2 ng mL^−1^ for all detection modes. Error bars indicate standard deviations from triplicate experiments

The resultant graphs in Fig. 4 showed the areas of red and blue peaks at test lines as a function of antigen concentrations from three independent experiments. Both red and blue decay areas increased with increasing antigen concentrations. However, the degrees of decay areas depend on the type of colours developed on the test lines. For pLDH only samples, red decay areas were higher than blue ones (Fig. 4a), while the PfHRP2 only samples exhibited the opposite trend (Fig. 4c). To validate the efficacy of the assay, the limit of detection (LoD) was estimated by adopting a standard approach defined as an average plus three times the standard deviation (S_non-target_ + 3SD) of the blank sample signal. The LoD at which all red and blue signals were distinguishable from the blank sample signals was estimated to be 31.2 ng mL^−1^ in all detection scenarios (inserted figures in Fig. 4).

**Fig. 5:**
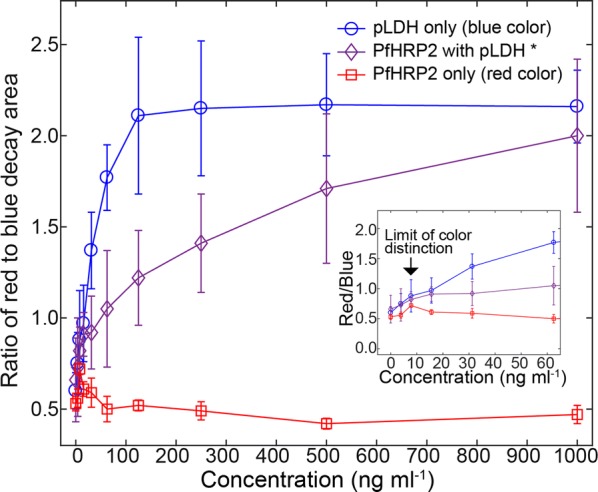
Ratios of red to blue decay areas. The simple function of the colour ratios calculated from Fig. 4 provided a criterion to discriminate colours as a function of sample concentrations. The blue curve with circle makers was above the other curves, indicating strong blue colours. For the blue curve, pLDH concentrations were shown in the x-axis. The middle curve with diamond markers was an intermediate curve in between top and bottom curves, and represented the mixture colours of red and blue. For the middle curve, PfHRP2 concentrations were shown in the x-axis, and pLDH/PfHRP2 was mixed at the ratio of 1:6. The bottom curve indicated the strong red colours. For the red curve, PfHRP2 concentrations were shown in the x-axis. The arrows in the inserted graph showed the limit of colour distinction from which the blue colours were distinguishable from the red colours. Error bars are standard deviations from triplicate experiments

Next, the ratio of decay areas of the red to blue was calculated to provide a simple method of color discrimination (Fig. 5). As expected, the decay ratios increased with increasing pLDH concentrations that attributed red colour intensities (top curve in Fig. 5). The region above the top blue curve is the pLDH only region, indicating *P. falciparum* negative.

By contrast, the ratio values decreased with increasing PfHRP2 concentrations (bottom curve in Fig. 5). The region below the bottom red curve contains PfHRP2 only. Since pLDH is pan-specific, it should always be present for malaria positive cases. For all of four human malaria species, the result will not fall into the PfHRP2 only region.

The decay ratios in the simultaneous detection were intermediate and included in between the top curve and bottom curve in Fig. 5, indicating it should be a mixture colour of blue and red. The region between the top blue curve and the bottom red curve contains both pLDH and PfHRP2, indicating *P. falciparum* positive.

The limit of colour distinction where the red and blue colours were distinguishable using the same definition of LoD was estimated. It can be observed that the top curve in Fig. 5 was always higher than the values plus 3SD of the bottom curve after 7.8 mg mL^−1^, set as the limit of colour distinction (inserted figure in Fig. 5).

### Validation of two-colour LFA by testing clinical samples

For the 15 negative samples tested, the colour intensities are below the LoD for both pLDH and PfHRP2 and therefore are regarded as malaria negative. To distinguish infection types and estimate antigen concentrations for the 10 malaria positive samples, colour discrimination was conducted with the RGB values from ImageJ analysis. Since pLDH is pan-specific and binds to all malaria species, the presence of pLDH can be expected in all malaria positive samples. The pLDH concentration can be estimated by its corresponding red decay areas with the calibration curve in Fig. 4. For all the malaria positive samples, a four-step trial and error method was adopted to determine whether the sample is *P. falciparum* or non-*P. falciparum* (i.e., *P. vivax*, *P. ovale*, or *P. malariae*).

- Step 1: ImageJ is being used for calculating the red and blue decay areas for the test line, then the ratio of red to blue decay areas was calculated and defined as ratio_cal.
- Step 2: Since pLDH is present in all positive clinical samples, assume the sample contains pLDH only and correlate the red decay areas measured in Step 1 with pLDH concentrations, with the calibration curve in Fig. 4a.
- Step 3: Find the value of the ratio of red to blue decay areas (y-axis) on the top blue curve in Fig. 5 at the calculated pLDH concentration (from Step 2), and define it as ratio_ref.
- Step 4: will consist of two scenarios: (1) If ratio_cal ≥ ratio_ref, then the sample falls into the region above the top blue curve in Fig. 5 (pLDH only region), indicating non-*P. falciparum,* but malaria positive (*P. vivax*, *P. ovale*, or *P. malariae*). In this scenario, the estimated pLDH concentrations from Step 2 are valid since the samples contain pLDH only; (2) If ratio_cal < ratio_ref, then the sample falls into the region between the top blue curve and the bottom red curve in Fig. 5 (pLDH + PfHRP2 region), indicating *P. falciparum* positive. In this scenario, the estimated pLDH concentrations from Step 2 are not valid since the samples contain pLDH and PfHRP2, and the calibration curve in Fig. 4a cannot be applied. The interpretation of mixture color was complicated, and possibly biased by image contrast. It also was not easy to create standard curves that can cover every scenario of color combinations.

The red and blue decay areas and colour ratios of the *P. falciparum* positive and *P. vivax* positive samples were presented in Tables 1, 2, respectively.

**Table 1:** Results of image analysis, and estimation of antigen concentrations and type of malaria infection from *Plasmodium falciparum* positive clinical samples

Sample no.	25	460	463	468	770
Microscopy examination	Positive *P. falciparum*	Positive *P. falciparum*	Positive *P. falciparum*	Positive *P. falciparum*	Positive *P. falciparum*
Red decay area at test line	917.11	1206.18	1335.83	1183.03	799.466
Blue decay area at test line	499.20	627.21	663.44	614.65	448.40
Red/blue ratio of clinical samples (ratio_cal)	1.84	1.92	2.01	1.92	1.78
Estimated red/blue ratio at the red decay area (ratio_ref)	1.95	2.13	2.14	2.12	1.83
LFA colour discrimination	Positive *P. falciparum*	Positive *P. falciparum*	Positive *P. falciparum*	Positive *P. falciparum*	Positive *P. falciparum*
ELISA results (pLDH: μg mL^−1^; PfHRP2: ng mL^−1^)	pLDH: 56.10 PfHRP2: 66.38	pLDH: * PfHRP2: > 312.5	pLDH: 20.12 PfHRP2: > 312.5	pLDH: 22.93 PfHRP2: > 312.5	pLDH: 67.74 PfHRP2: 240.65

* > 5.00 μg mL^−1^ but < 20.00 μg mL^−1^

**Table 2:** Results of image analysis, and estimation of antigen concentrations and type of malaria infection from *Plasmodium vivax* clinical samples

Sample no.	471	442	486	457	476
Microscopy examination	Positive *P. vivax*	Positive *P. vivax*	Positive *P. vivax*	Positive *P. vivax*	Positive *P. vivax*
Red decay area at test line	631.10	846.20	826.30	907.39	857.67
Blue decay area at test line	299.22	416.34	356.00	445.82	409.38
Red/blue ratio of clinical samples (ratio_cal)	2.11	2.03	2.32	2.05	2.10
Estimated red/blue ratio at the red decay area (ratio_ref)	1.63	1.88	1.86	1.94	1.89
LFA colour discrimination	Malaria positive, *P. falciparum* negative	Malaria positive, *P. falciparum* negative	Malaria positive, *P. falciparum* negative	Malaria positive, *P. falciparum* negative	Malaria positive, *P. falciparum* negative
LFA results (pLDH: μg mL^−1^)	pLDH: 1.02	pLDH: 1.63	pLDH: 1.56	pLDH: 1.86	pLDH: 1.67
ELISA results (pLDH: μg mL^−1^; PfHRP2: ng mL^−1^)	pLDH: 2.70 PfHRP2: negative	pLDH: * PfHRP2: negative	pLDH: * PfHRP2: 3.35	pLDH: * PfHRP2: negative	pLDH: * PfHRP2: negative

* > 5.00 μg mL^−1^ but < 20.00 μg mL^−1^

As shown in Table 1, the 5 samples were confirmed as *P. falciparum* positive by microscopy examination. Using the four-step trial and error method illustrated above, the red to blue reference ratio (ratio_ref) was estimated at the corresponding red decay area by the pLDH calibration curves and compared with the red to blue ratio of clinical samples (ratio_cal). Since ratio_cal < ratio_ref for all the 5 samples listed in Table 1, all the samples fall into scenario two in Step 4 as described above. Then it could be concluded that the sample is *P. falciparum* positive. For example, sample No. 25 is confirmed as *P. falciparum* positive by microscopy. The red decay area and blue decay area at test line are 917.11 and 499.20, respectively. The red to blue ratio of sample No. 25 (ratio_cal) is therefore 1.84. The red to blue reference ratio at the corresponding red decay area (917.11) (ratio_ref) is calculated by the pLDH calibration curve in Fig. 4a, and the value is 1.95. Since 1.95 (ratio_ref) > 1.84 (ratio_cal), then sample No. 25 is confirmed as *P. falciparum* positive by LFA colour discrimination. This agrees with both microscopy and ELISA results.

As shown in Table 2, the 5 samples were confirmed as *P. vivax* positive by microscopy examination. Similarly, ratio_ref and ratio_cal were obtained. Since ratio_cal > ratio_ref for all the 5 samples listed in Table 2, all samples fall into the pLDH only region, indicating malaria positive but negative for *P. falciparum*. In this case, the pLDH calibration curve in Fig. 4a could be applied to get the pLDH concentrations for all the samples in Table 2. For example, sample No. 471 is confirmed as *P. vivax* positive by microscopy. The red decay area and blue decay area at test line are 631.10 and 299.22, respectively. The red to blue ratio of sample No. 471 (ratio_cal) is therefore 2.11. The red to blue reference ratio at the corresponding red decay area (631.10) (ratio_ref) is calculated by the pLDH calibration curve in Fig. 4a, and the value is 1.63. Since 2.11 (ratio_cal) > 1.63 (ratio_ ref), then sample No. 471 is confirmed as malaria positive but *P. falciparum* negative by LFA colour discrimination. This agrees with both microscopy and ELISA results.

For all the samples in Table 2, it should be noted that the pLDH quantification results showed discordance between the LFA and ELISA methods. The estimated concentration in LFA was lower than that of ELISA. This error could be attributed to the difference in standard curves for buffer and whole blood clinical sample [18, 19]. It should also be noted for sample No. 486, PfHRP2 concentrations with LFA and ELISA methods are 0 and 3.35 ng mL^−1^, respectively, since 3.35 ng mL^−1^ is already beyond the LoD of LFA for PfHRP2 detection.

## Discussion

The multiplexed LFA that employs two different colours of latex particles for detecting pLDH and PfHRP2 simultaneously at a single test line was demonstrated. The colour developed at the test regions varies from antigen types and concentrations. The assay was capable of distinguishing malaria positive from malaria negative samples. A colour discrimination protocol was also developed for discriminating malaria species if the assay was proved malaria positive.

The assay was first validated by testing recombinant malaria antigens in washing buffer. Based on the buffer test, the LoD for the assay to differentiate both pLDH and PfHRP2 from blank samples was defined, to determine whether a sample is malaria positive or negative. The LoD of the LFA was 31.2 ng mL^−1^ for both pLDH and PfHRP2, and this performance was similar to that of conventional gold nanoparticle LFAs [20].

The limit of colour discrimination to differentiate pLDH from PfHRP2 was also defined, to differentiate *P. falciparum* infection from the other three human malaria species (*P. vivax*, *P. ovale*, and *P. malariae*), for malaria positive samples. The limit of colour discrimination of the assay in buffer test was 7.8 ng mL^−1^.

Twenty-five malaria clinical samples in whole blood were tested and the assay performance was validated by ELISA and microscopy results. For the clinical samples tested, 5 samples showed *P. falciparum* positive, 5 samples showed *P. falciparum* negative but contained other malaria species, and 15 samples showed malaria negative, which agreed with ELISA and microscopy results. The pLDH concentrations for *P. vivax* positive clinical samples were also quantified and compared with ELISA results. The discrepancies could be attributed to the differences in standard curves for buffer and whole blood samples.

## Conclusion

A two-colour LFA to differentiate *P. falciparum* infection from the other three human malaria species was developed, with a single test line on the strips with pre-coded colours for different analytes. Co-infections of different malaria species, though uncommon, need to be further examined to distinguish from the single infection of *P. falciparum*. For the practical use in peripheral settings, the image analysis algorithm can be implemented in the image reader [21, 22]. It is envisioned that the two-colour LFA can be further extended to a three-colour system by incorporating red, blue and green conjugations together, and this offers the possibility to detect 6 different analytes with two test lines on the strip. The two-colour LFA provides a simple approach to overcoming the physical limitations of traditional LFAs, presenting a feasible method for multiplexing.

## Data Availability

All data generated or analysed during this study are included in this published article.
